# The Parent Attitudes about Childhood Vaccination against COVID-19 in Tunisia

**DOI:** 10.1192/j.eurpsy.2024.1068

**Published:** 2024-08-27

**Authors:** I. Bouguerra, A. Touiti, W. Askri, S. Hlayem, A. Bouden

**Affiliations:** Child and adolescent psychiatry, Razi Hopsital, Manouba, Tunisia

## Abstract

**Introduction:**

The vaccination of children and adolescents against covid 19 is an ongoing debate. While in some countries the program of vaccination of children under 12 years old is already implemented, in others the balance of risk and benefits is a dilemma. Parents’ perception and decision about covid vaccination is an important parameter to consider.

**Objectives:**

The ain of this study is to evaluate the parents’ attitude about childhood vaccination against Covid 19 in Tunisia.

**Methods:**

The “vaccine hesitancy scale (VHS) adopted from WHO’s Strategic Advisory Group of Immunization” with 8 items translated in tunisian dialect and an additional ten survey idem about the Characteristics of parents and their vaccination status against Covid 19. The survey is distributed on social media groups of parents of tunisian children and adolescents.

**Results:**

Thirty parents have answered the survey.More than 80% of the participants were female with an average age of 38 years old.Among 30 participants 24 were vaccinated against covid 19 at least for once, but only one of them 10% decided to vaccinate their children against covid 19,while 3% are indecisive about the subject.Although 97 % of the children and adolescents have already been vaccinated completely according to the national vaccination program. The most common reasons for the refusal were.Parents consider that routine childhood vaccines are safe, necessary and useful more than covid vaccine. Some parents reported that their children have been infected by the virus so they have doubts about the usefulness of the vaccination.

**Conclusions:**

A year after the pandemic, covid 19 contamination and vaccination against the virus are still an issue. With the emergence of new variants, the decline in protective measures, vaccination against covid is in the process of integrating routine programs. But the lack of information on the effectiveness of the vaccine and the adverse effects are a source of hesitation and refusal for parents. a large-scale national study is necessary before.

**Disclosure of Interest:**

None Declared

